# Host and viral mechanisms of congenital Zika syndrome

**DOI:** 10.1080/21505594.2019.1656503

**Published:** 2019-08-26

**Authors:** Brooke Liang, José Paulo Guida, Maria Laura Costa, Indira U. Mysorekar

**Affiliations:** a Department of Obstetrics and Gynecology, Washington University School of Medicine, St. Louis, MO, USA; b Department of Obstetrics and Gynecology, School of Medical Sciences, University of Campinas, Campinas, Brazil; c Department of Pathology and Immunology, Washington University School of Medicine, St. Louis, MO, USA; d Center for Reproductive Health Sciences, Washington University School of Medicine, St. Louis, MO, USA

**Keywords:** Placenta, trophoblast, hydroxychloroquine, autophagy, type I interferon, interferon lambda

## Abstract

In 2015–2016, in the Americas, and especially in northeast Brazil, a significant number of cases of microcephaly and other congenital brain abnormalities were linked with an outbreak of Zika virus (ZIKV) infection in pregnant women. While maternal symptoms of ZIKV are generally mild and self-limiting, clinical presentation in fetuses and newborns infected is extensive and includes microcephaly, decreased cortical development, atrophy and hypoplasia of the cerebellum and cerebellar vermis, arthrogryposis, and polyhydramnios. The term congenital ZIKV syndrome (CZS) was introduced to describe the range of findings associated with maternal-fetal ZIKV transmission. ZIKV is primarily transmitted by *Aedes aegypti* mosquitoes, however non-vector-dependent routes are also possible. Mechanisms of maternal-fetal transmission remain unknown, and the trans-placental route has been extensively studied in animal models and in human samples. The aim of this review was to summarize recent studies that helped to elucidate the mechanism of CZS in animal models and observational studies. There are still challenges in the diagnosis and prevention of CZS in humans, due to the large gap that remains in translating ZIKV research to clinical practice. Translational research linking governments, local health workers, scientists and industry is fundamental to improve care for mothers and children.

## Introduction

Zika virus (ZIKV) transitioned from a generally unknown entity to one of the most studied viruses worldwide over a one-and-a-half-year period. In 2015–2016, the virus was responsible for an outbreak in the Americas and especially in Brazil. During this outbreak, ZIKV infections in pregnant women were linked for the first time to severe fetal malformations, with an impressive number of cases of microcephaly and other congenital brain abnormalities. In 2015–2016, Brazil reported more than 200,000 probable ZIKV cases and nearly 2,000 cases of microcephaly [1–6]. Due to rapid spread and high numbers of suspected cases, ZIKV infection was declared a Public Health Emergency of International Concern on 1 February 2016 [7]. Soon after, in March 2016, the World Health Organization announced there was scientific consensus causally linking congenital brain abnormalities to ZIKV infection [8,9].

ZIKV is a flavivirus similar to Dengue, West Nile, yellow fever, and Japanese encephalitis viruses. It is an arbovirus transmitted primarily by *Aedes aegypti* mosquitoes and was first identified in humans in Uganda around 1952. Between 1952 and 2015 there were two major outbreaks. The first was in Yap, Micronesia in 2007 and the second was in French Polynesia in 2013 [10,11]. ZIKV can also be transmitted to humans through non-vector-dependent routes, including sexual transmission, blood transfusion, or maternal-fetal transmission [12].

ZIKV is a member of the TORCH family, which includes Toxoplasma gondii, rubella virus, cytomegalovirus, and herpes simplex virus. This family of pathogens possesses the relatively unusual ability to transmit from a mother to her developing fetus or newborn [13]. Precise mechanisms of maternal-fetal transmission during pregnancy remain largely unknown. Hypothesized routes of trans-placental ZIKV transmission include direct infection of the SYN layer, infection of extravillous trophoblasts (EVTs) (cells that anchor the placenta to the uterine wall), infection of the decidua and/or maternal microvasculature, and infection of feto-placental macrophages (Hofbauer cells) [14–17].

The term congenital ZIKV syndrome (CZS) has been adopted recently to describe the range of findings associated with maternal-fetal ZIKV transmission [18]. While pregnant women infected with ZIKV often reported no symptoms or only self-limiting flu-like symptoms, a broad spectrum of clinical presentations has been reported in fetuses and newborns of women infected with ZIKV during pregnancy. Although microcephaly has been the hallmark finding in these fetuses and newborns, not all affected fetuses demonstrated microcephaly. Other clinical signs of CZS include decreased cortical development and atrophy and hypoplasia of the cerebellum and cerebellar vermis. Arthrogryposis and polyhydramnios, likely due to swallowing impairment because of brain injury, are also common findings [19,20].

The rapid spread of the ZIKV epidemic and the devastating congenital defects it produced spurred governments, the healthcare industry, academia, physicians, and patient advocates internationally. Vast amounts of knowledge have been obtained on the viral genome, structure, pathogenesis and mechanisms of maternal-fetal transmission of ZIKV. Studies continue to address clinical features, fetal complications, long-term consequences of the infection in humans, as well as routes of infection and pathophysiology of the disease in animal models [21,22]. Specific interactions between host and environmental factors, differences among strains, the role of co-infections, differences in placental infection according to gestational age of infection, and long-term effects in exposed fetuses born apparently normal remain largely unknown. Of note, there still exists no treatment or vaccine for ZIKV infection or CZS that has been approved for use in patients. This is despite extensive work in the development of vaccines and neutralizing antibodies, which has been covered in excellent recent reviews [18,23–26].

In this review, we focus on a number of recent studies that have helped elucidate the mechanism of CZS using animal models and observational studies in humans, and we reflect on lessons learned from the epidemic now that it has waned.

## Placental host defenses and ZIKV

The placenta is the primary organ responsible for nurturing the fetus during development. In human placentas, fetal-derived anchoring and chorionic villi form connections to maternal structures. Anchoring villi possess specialized cells, extravillous trophoblasts (EVTs), at their distal ends. These extravillous trophoblasts specifically invade the maternal decidua basalis and maternal vasculature. Chorionic villi are branching tree-like structures that are bathed in maternal blood of the intervillous space. At the core of these chorionic villi are blood vessels that connect to fetal circulation. The surface chorionic villi is composed of an inner layer of cytotrophoblast (CTB) cells and an outer layer of syncytiotrophoblast (STB) cells, and it mediates exchange of materials between maternal and fetal circulations [27,28]. Other cell types present in the placenta include stromal cells and immune cells.

The mouse placenta can be divided into three layers. The outermost layer is the maternal compartment which includes the decidua basalis. The middle layer, referred to as the junctional zone, facilitates placental attachment to the uterus. It contains spongiotrophoblasts, a type of cytotrophoblast, and trophoblast giant cells, which invade the decidua basalis. The inner layer, the labyrinth zone, is the site of nutrient and gas exchange between maternal and fetal compartments. Here, fetal capillaries, lined by fetal endothelial cells, are separated from maternal sinusoids by a layer of mononuclear trophoblasts and a bilayer of multinucleated syncytiotrophoblasts [29,30]. Although mouse and human placentas differ in many aspects, both constitute effective barriers against pathogenic insults to the developing fetus.

To understand how ZIKV overcomes placental host defenses, mouse models were developed. Initially, efforts to study ZIKV vertical transmission in a mouse model were hindered by ZIKV’s inability to infect wild-type mice [31]. This challenge was overcome after it was discovered that mice lacking an intact interferon signaling response were susceptible to ZIKV infection as evidenced by weight loss, decreased survival, and neurologic disease [31]. The interferon signaling pathway, a major component of antiviral host defense, proved to be critical to the difference between mouse and human susceptibilities to ZIKV as the NS5 protein of ZIKV binds to and facilitates degradation of human STAT2, which participates in signal transduction downstream of the interferon α/β receptor, but not mouse STAT2 [32,33].

Initial studies in mice exploring ZIKV vertical transmission utilized mice lacking the interferon α/β receptor (*Ifnar1*^−/-^ mice) or WT mice treated with a monoclonal antibody against the interferon α/β receptor. A trans-placental route of infection was strongly suggested in this model by staining of placental tissues which revealed ZIKV particles in placental trophoblasts and adjacent fetal endothelial cells. Moreover, apoptosis of trophoblasts, disruption of fetal capillaries, and increased nucleated fetal erythrocytes were visualized, suggesting impairment of basic placental functions with ZIKV infection [34]. Studies utilized *Ifnar1*^−/-^ females crossed to *Ifnar1*^±^ males and noted that ZIKV titers were higher in placentas of *Ifnar1*^−/-^ offspring, whereas *Ifnar1*^±^ fetuses were resorbed due to abnormal placental labyrinth development in the presence of type I interferon [35,36]. Thus, interferon α/β signaling in the placenta is an important component of host defense against ZIKV infection.

Type III interferon, namely Interferon-λ has also been implicated in ZIKV infection of the placenta and the female reproductive tract [37–40]. In one model, mice lacking the interferon-λ receptor demonstrated maintained susceptibility to ZIKV-mediated disease later in gestation, unlike previous interferon-deficient models which showed decreased fetal and placental disease with increased gestational age at infection [39]. These studies show that mice lacking any component of the interferon signaling pathway are susceptible to vertical transmission of ZIKV and adverse fetal outcomes.

Mouse models have also been used to study sexual transmission of ZIKV. WT female mice vaginally inoculated with ZIKV were shown to have persistence of local ZIKV proliferation, and this effect was exaggerated in mice lacking the interferon α/β receptor or IRF3 and IRF7 (transcription factors involved in the innate immune response upstream of interferon) [41]. Furthermore, vaginal inoculation of ZIKV early during pregnancy (at embryonic day 4.5) was associated with intrauterine growth restriction and fetal resorptions in WT females crossed with WT males, Ifnar1^−/-^ females crossed with WT males, and IRF3^−/-^IRF7^−/-^ females crossed with IRF3^−/-^IRF7^−/-^ males. ZIKV titers were detected in fetuses and placentas of Ifnar1^−/-^ females crossed with WT males and IRF3^−/-^IRF7^−/-^ females crossed with IRF3^−/-^IRF7^−/-^ males. While ZIKV titers were not detected in fetuses of WT females, electron microscopy showed evidence of ZIKV infection in fetal brains from infected WT females [41]. Further studies demonstrated that ZIKV-infected Ifnar1^−/-^ male mice were capable of transmitting ZIKV to naïve Ifnar1^−/-^ female mice, as evidenced by growth restriction of their fetuses, ZIKV particles observed on electron microscopy of fetal brains, and anti-ZIKV antibodies found in the sera of females treated with medroxyprogesterone acetate [42]. Together, these studies demonstrate sexual transmission of ZIKV in mouse models and its potential to couple with vertical transmission and cause fetal disease.

Recently, mouse models have been developed to tackle the problem of studying ZIKV only in artificially immunocompromised states. One model employed direct intrauterine inoculation of ZIKV in an immunocompetent mouse. This model recapitulated fetal neural disease associated with ZIKV, worse outcomes associated with infection at earlier gestational age, and the presence of ZIKV in trophoblasts and fetal endothelial cells, and further demonstrated activation of the type I interferon signaling pathway with ZIKV infection [43]. Another model administered a large ZIKV viral load to WT mice and recapitulated adverse fetal outcomes [44]. A knock-in model sought to replicate human vulnerability to ZIKV in mice by introducing humanized STAT2 to the mouse STAT2 locus. This humanized model demonstrated maternal infection, placental infection, and vertical transmission unlike wild-type mice [45].

Additional models were used to investigate ZIKV viral entry into cells of the maternal-fetal interface. The TAM family of receptors (Tyro3, Axl, and Mertk) are known sites of entry exploited by flaviviruses. However, mice lacking TAM receptors did not show a decrease in ZIKV maternal infection or adverse fetal effects [39]. TIM1 is another receptor or factor facilitating viral entry that is ubiquitously expressed in placental tissues. In one study, duramycin, a TIM1 inhibitor, demonstrated greater ZIKV inhibition than an Axl inhibitor, suggesting a role for TIM1 in ZIKV vertical transmission [14]. Mechanisms underlying ZIKV viral entry *in vivo* into the placenta remains an open avenue of investigation. .

Innate immune mechanisms in the context of congenital ZIKV infection have been investigated. Inhibition of toll-like receptors (TLRs) 3 and 8 were recently shown to inhibit the cytokine output of ZIKV-infected trophoblasts [46]. Inflammatory cytokines resulting from placental infection have been associated with congenital diseases in the past, and Luo et al. argue that it is the maternal inflammation produced in response to ZIKV infection rather than ZIKV tropism for trophoblasts that is responsible for CZS, as the other flaviviruses dengue and yellow fever showed similar tropism without causing congenital disease [46]. Further studies such as Novak et al. [47] are required to establish the independent effect of inflammatory cytokines and chemokines on fetal development.

The autophagy pathway functions to catabolize intracellular units to produce energy and structural components. Autophagy is recognized to be a key player in host defense through xenophagy, the lysosomal degradation of intracellular pathogens. Autophagy has also been shown to be important in placental defense against pathogens [48,49]. However, flaviviruses are known to co-opt the autophagy pathway to enhance their intracellular replication and mitigate viral-mediated stress [50]. Indeed, a wide variety of flaviviruses, such as hepatitis C virus, dengue virus, Japanese encephalitis virus, West Nile virus appear to subvert autophagy to promote their survival. In the context of CZS, Cao et al. demonstrated that ZIKV increases autophagic flux in infected mouse placentas. Conversely, inhibition of autophagy through a knockout model of a key autophagy gene, *atg16l1*, important for autophagosome formation inhibited placental ZIKV infection and vertical transmission in a mouse model [48,51]. Furthermore, Cao et al., demonstrated that *atg16l1* deficiency exclusively in the placenta was sufficient to limit ZIKV infection and vertical transmission. Loss of ZIKV-induced autophagy in the placenta rescued intrauterine growth restriction and limited viral load in fetal brain. Thus, the autophagy pathway activity has physiological significance for determining the outcomes of congenital ZIKV syndrome [52–55]. Further work is needed to elucidate how ZIKV co-opt autophagy.

## Viral mechanisms contributing to CZS

Microcephaly was the first fetal abnormality linked with congenital infection by ZIKV. Subsequently severe cerebral atrophy, ventriculomegaly, and intracranial calcification were also identified, expanding the spectrum of fetal malformations associated with ZIKV infection.

Animal models showed that ZIKV infection during pregnancy results in reduced fetal cerebral cortical surface area. It was hypothesized that neural progenitor cells are specifically targeted by the virus. Following infection, ZIKV has an inhibitory effect on the proliferation of these cells and this initiates the sequence of malformations associated with CZS [56].

Another study involving animal models used ZIKV isolated from a febrile case from Brazil to demonstrate a reduced number of cells and thickness of cortical layers. This study also used an *in vitro* model based on neurospheres and cerebral organoids and showed that these structures, when infected with a Brazilian strain of ZIKV, are smaller than uninfected controls and controls infected with an African strain of ZIKV [57]. Neurospheres and cerebral organoids are structures made of neural stem cells that simulate tissue responses to various experimental conditions [58,59]. In line with the Brazilian study, other studies [60,61] showed similar findings using neurospheres and ZIKV infection, further suggesting that the observed cortical thinning is due to ZIKV effects on neural progenitor cells. Of note, the destruction of these cells contributes significantly to the clinical phenotype of CZS.

Over the past two years, many studies have investigated ZIKV structure and function to better understand how the virus overcomes placental defenses. ZIKV, like other flaviviruses, possesses an approximately 11-kb single-stranded, positive-sense RNA genome. Translation of this genome produces a single viral polypeptide, which is cleaved by viral and host proteases into functional proteins. The ZIKV genome encodes three structural proteins (capsid, pre-membrane, and envelope), and seven nonstructural proteins (NS1, NS2A, NS2B, NS3, NS4A, NS4B, and NS5) [62,63]. A number of these proteins have been targeted for developing or repurposing drugs against ZIKV.

Drug repurposing is an attractive strategy in the face of an epidemic, as an existing drug can be quickly put into practice whereas development of a new drug or vaccine may not be completed before the epidemic reaches the end of its natural course. Several studies screened large libraries of existing drugs for candidates to test against ZIKV. For example, Xu et al. identified a caspase inhibitor, emricasan, and several inhibitors of cyclin-dependent kinases that demonstrated neuroprotection during ZIKV infection [64]. Barrows et al. identified known anti-flaviviral drugs (e.g. bortezomib and mycophenolate) and drugs without known antiviral properties (e.g. daptomycin) as having anti-ZIKV activity in a number of human cell types, including placenta and neural stem cell [65]. Rausch et al. identified nanchangmycin as a ZIKV entry inhibitor across cell types [66]. Bullard-Feibelman et al. focused their assays on sofosbuvir, an RNA-dependent RNA polymerase inhibitor used to treat chronic hepatitis C, a flavivirus distantly related to ZIKV. Sofosbuvir protected cells in culture from multiple strains of ZIKV, it protected human-derived neural stem cells from ZIKV, and it reduced mortality rate in interferon-deficient mice inoculated with a mouse-adapted ZIKV strain [67]. Chan et al. identified bromocriptine, a dopamine agonist, as an effective inhibitor of ZIKV *in vitro*, through binding and inhibition of the ZIKV NS2B-NS3 protease [68].

Other groups focused their studies on drugs with existing approved uses during pregnancy. Viral and host mechanisms can intersect to facilitate ZIKV vertical transmission and thus offer promising points of therapeutic intervention. An excellent example of this is hydroxycholoroquine (clinically prescribed under the brand name Plaquenil). Hydroxychloroquine is an antimalarial and anti-rheumatic drug also known to inhibit autophagy. Importantly, Cao et al., demonstrated that pharmacologic inhibition of autophagy with hydroxychloroquine attenuated ZIKV infection in mouse placentas and ameliorated fetal growth restriction associated with ZIKV [51]. The mechanism for ZIKV upregulation of autophagy was elucidated by Liang et al., who showed that the ZIKV proteins NS4A and NS4B cooperatively dysregulate the Akt-mTOR pathway leading to increased autophagy. This effect was not observed with the NS4A and NS4B proteins of the related flavivirus, dengue [69]. Hydroxychloroquine was the top hit in an *in silico* screen using a US Food and Drug Administration (FDA)-approved drug library for candidates that target the NS2B-NS3 protease of ZIKV [70]. Using molecular docking, molecular dynamics simulations, and enzyme kinetic studies, Kumar et al., demonstrated that hydroxychloroquine has high binding affinity for the active site of the NS2B-NS3 protease. Hydroxychloroquine’s anti-ZIKV activity was subsequently demonstrated in placental cells in culture. Shiryaev et al. also identified chloroquine as capable of attenuating neural disease and vertical transmission of ZIKV in a mouse model [71]. As hydroxychloroquine is already approved for chronic use throughout human pregnancy for women with systemic lupus erythematosus and other rheumatologic conditions [72–75], it is posited as a promising option for human trials.

## Challenges in the diagnosis and prevention of CZS in humans

Despite the tremendous progress in cell culture and animal model work reviewed here and elsewhere [23,76–85], there remains a large gap in the translation of ZIKV research from bench to bedside. One initial challenge has been defining the disease pathologically. Case reports and case series have described mild and nonspecific placental pathologic findings in ZIKV-affected pregnancies. Pathology reports included descriptors such as chronic placentitis, chronic villitis, increased Hofbauer cells, variable perivillous fibrin and mononuclear cells, villous immaturity, stromal fibrosis and calcification, increased vascularity, lymphocytic deciduitis and focal syncytiotrophoblast necrosis [77,86–88].

It has also been a challenge to standardize the diagnosis of CZS. Examination of placentas from suspected or confirmed cases of ZIKV infection is recommended as part of gold-standard care for women and their newborns [89]. However, protocols and standards for placental sample collection and storage have not been widely adopted and are often not precisely worded. The official Brazilian Ministry of Health guideline, for example, has no figures to guide systematic placental sampling and only a statement that 3 fragments, 1.0 × 1.0cm, be obtained with no recommendation on depth or sites of collection expected [90]. Moreover, not all medical facilities possess the infrastructure required to perform detailed placental pathological analyses.

Correlating gestational age at infection with CZS phenotype has been challenging. Most detailed cases represented first trimester infections with symptomatic disease, which were associated with significant numbers of abortions, stillbirths and neonatal deaths [77,86–88,91–95]. There have been reports of ZIKV inducing fetal disease and/or adverse pregnancy outcomes with maternal infections well beyond the first trimester [3,96], however, poorer outcomes are expected when the infection occurs earlier in gestation and in particular during organogenesis. Multiple arboviruses (such as dengue virus and yellow fever virus) that produce similar clinical presentations are endemic to areas affected by ZIKV [97]. Many symptomatic people do not seek medical care unless they manifest severe features, which might present after the optimal timing for sample collection and diagnosis has passed.

An important consideration is the worldwide variation in antenatal screening availability and management options for women with fetal congenital abnormalities. Of note, it is illegal or highly restricted to obtain an induced abortion in most Latin American countries, including Brazil [98]. These factors help explain the sparsity of tissue samples and ultrasound images from earlier gestational ages. Another finding that must be addressed is the existence of placental tissue that tested positive for ZIKV infection in apparently unaffected neonates [98]. Detection of ZIKV RNA in the placenta does not discriminate between maternal and fetal infection. Therefore, questions arise as to whether certain infants were protected by an effective immune response, whether placentas protect against ZIKV more effectively during later gestation, whether more advanced stages of organogenesis are immune to ZIKV-related disturbances, and whether these neonates will continue to appear normal throughout childhood development. These questions will need to be addressed through long-term follow-up of infants and through experimental studies involving animal models and *in vitro* work.

As of the end of 2018, a valiant cross-disciplinary effort has provided us with incredible insights into the mechanism of CZS. The epidemic has now waned, stalling clinical trials for many drugs and new vaccines that have been proposed for preventing ZIKV infection and CZS. This review highlights key knowledge that has been gained regarding the mechanism of CZS but also points to clinical challenges surrounding CZS that have not been adequately addressed. With climate change and increasing awareness of the potential for arboviruses to undergo maternal-fetal transmission [99,100], further translational research, especially in collaboration with governments, local health workers, and the pharmaceutical industry, is needed to better equip us to face future challenges to maternal-fetal health.
